# Exploring DNA quality of single cells for genome analysis with simultaneous whole-genome amplification

**DOI:** 10.1038/s41598-018-25895-7

**Published:** 2018-05-10

**Authors:** Christiane Bäumer, Evelyn Fisch, Holger Wedler, Frank Reinecke, Christian Korfhage

**Affiliations:** 1 QIAGEN GmbH, Department for Research & Foundation, QIAGEN-Strasse 1, 40724 Hilden, Germany; 2 QIAGEN GmbH, Department for NGS, PCR-Array and WGA-Service, QIAGEN-Strasse 1, 40724 Hilden, Germany

## Abstract

Single cell genome analysis methods are powerful tools to define features of single cells and to identify differences between them. Since the DNA amount of a single cell is very limited, cellular DNA usually needs to be amplified by whole-genome amplification before being subjected to further analysis. A single nucleus only contains two haploid genomes. Thus, any DNA damage that prevents amplification results in loss of damaged DNA sites and induces an amplification bias. Therefore, the assessment of single cell DNA quality is urgently required. As of today, there is no simple method to determine the quality of a single cell DNA in a manner that will still retain the entire cellular DNA for amplification and downstream analysis. Here, we describe a method for whole-genome amplification with simultaneous quality control of single cell DNA by using a competitive spike-in DNA template.

## Introduction

Single cell genome analysis has become increasingly important and has rapidly evolved over the past decade. Two major motivations focus genome analysis on single cells. (1) Samples may comprise a very small number of cells or even a single cell and there is no choice to use larger samples^1,2^. (2) Other samples comprise cells of high genomic variation. Cell heterogeneity plays a central role in biological phenomena during normal development or disease (e.g., brain development, cancer, or aging)^3–6^. In recent years, it has become apparent that cells can acquire genome changes (e.g. mutations, copy number variations (CNV), chromosomal aberrations) that may be propagated to daughter cells and results in mosaics of cells with different genotypes^3,4^. Originally caused by a few genomic mutations, multiple changes in single cells can result in altered cell programming and cell division rate. To find the clonal development path of mosaic tissues, single cell genome analysis is a compelling requirement^4,7^.

To uncover genomic variation in individual cells, methods for deep genome analysis are necessary. These techniques include massively parallel sequencing (known as next generation sequencing, NGS), microarray analysis, or panel real-time PCR analysis. Typically, 1 ng to 1 μg of DNA is necessary, corresponding to the DNA amount of approximately 10^2^ to 10^5^ human cells. The DNA amount required for those genome analyses is at least 100-fold higher than the genome content of a single human cell (6 pg). Consequently, accurate amplification of the genomic DNA (whole genome amplification, WGA) is required for reliable genetic analysis. Whole-genome-amplification can generate large amounts from minute quantities of isolated DNA or even from single cells^8–11^. Incomplete or biased genome amplification with missing or underrepresented loci information is a frequently observed limitation when analyzing single cell genomes. Besides other factors, incomplete whole genome amplification is often a result of low template quality^12^. Genome damage (e.g. DNA breaks, abasic sites, UV induced thymine dimers, formalin modified bases etc.) can occur during cell treatment, harvesting, selection or cell storage. Most of the damaged DNA regions prevent the amplification process at the site of damage. We will refer to these sites as blocking sites or stop sites.

Different methods have been proposed to assess the quality of DNA samples prior to amplification. In the past decade, a couple of quality assays have been developed that address the integrity of DNA. Most of them are based on real-time PCR that quantifies the copy number of differently sized PCR products^13^. However, real-time PCR is limited to small amplicons and performs poorly when measuring DNA integrity over distances larger than 500 bp. Additionally, real-time PCR assays are limited to a small number of genomic loci which may behave differently compared to the whole genome. Most important, applying these methods results in the consumption of the single cell genome that would not be available for WGA and deep genome analysis. Therefore, none of these methods can be used for quality control of a single cell genome. Other methods use bioinformatic analysis and can be applied only after laborious and cost intensive microarray or NGS analysis^14^.

We have developed a new method that combines a quality assay of the single cell target DNA and whole-genome-amplification (WGA) for further downstream analysis. Here, we present a Control-DNA that is used as competitive spike-in control in single cell WGA reactions. The assay makes use of the preferential amplification of long DNA fragments by the Phi29 DNA polymerase. Consequently, fragment lengths or distances between polymerase stop sites of Control-DNA and single cell DNA are compared during the WGA reaction. The relative amplification rate of Control-DNA after WGA can be determined by real-time PCR and inversely correlates with the quality of single cell DNA and WGA DNA.

## Results

### Mechanism

**Figure 1:**
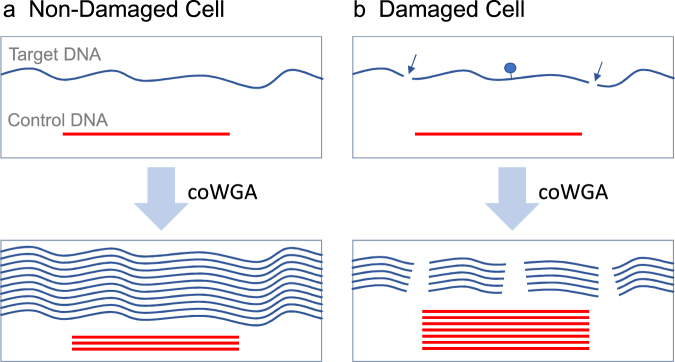
Competitive whole genome amplification (coWGA) of Control-DNA (red line) and single cell DNA (blue line). (**a**) In non-damaged cells, the double-stranded DNA is mostly intact and does contain very few blocking sites. Here, cellular DNA is longer than Control-DNA. In this case, the amplification of the large, unbroken double-stranded DNA out-competes the amplification of the Control-DNA. (**b**) In damaged cells, genomic DNA may have multiple breaks (arrows) and sites blocking Phi29 polymerase (blue dot). As a result, the amplification rate is reduced and the large sized Control-DNA outcompetes the amplification of the low quality cellular DNA. A high amount of Control-DNA indicates a low quality of cellular target DNA and vice versa.

Competitive whole genome amplification (coWGA) is based on multiple displacement amplification (MDA) using the DNA polymerase from *Bacillus subtilis* phage Phi29. The Phi29 polymerase is a highly processive polymerase with proofreading activity (3′-5′ exonuclease) and elongates primers up to 70,000 bp independently of sequence composition^15^. MDA results in whole genome amplification from tiny samples like a single cell with high genome coverage and low error rates due to the proofreading and strand-displacement activities of Phi29 polymerase^16,17^. Therefore, Phi29 polymerase is highly suitable for single cell WGA. Because of its processivity, Phi29 polymerase is sensitive to a high degree of template fragmentation and the presence of blocking sites, both of which will decrease amplification efficiency and increase amplification bias. To enable quality assessment of the target DNA of a single cell after whole genome amplification, a Control-DNA is spiked prior to the MDA reaction. Here, we used lambda phage DNA as competitive Control-DNA. For a better understanding of coWGA, two scenarios are described (see Fig. 1). If cellular DNA stems from a non-damaged cell, the double-stranded DNA contains only a very few blocking sites for DNA synthesis. In this case, the amplification of the large target DNA out-competes the amplification of the Control-DNA. As a result, the fraction of Control-DNA after amplification will be low. The representation of Control-DNA can be determined by real-time PCR. Therefore, high Cq values corresponds to a low amplification rate of Control-DNA and thereby to a high quality of the single cell DNA. In contrast, low-quality single cell DNA (characterized by a high number of blocking sites) will not amplify with high efficiency and the spiked Control-DNA outcompetes the amplification of cellular DNA during WGA reaction.

The method is comparable to competitive PCR, a method performing a simultaneous amplification of a spike-in DNA and the target DNA to quantify target DNA amount after PCR^18,19^. In contrast to competitive PCR, the amount as well as the amplifiable length of target DNA are the main parameters determined during coWGA. The efficiency of DNA amplification by Phi29 polymerase correlates with the length of target DNA present during the WGA reaction. Therefore, short DNA fragments or DNA fragments containing multiple DNA synthesis stop sites (e.g. abasic sites, thymine dimers) are outcompeted by a long Control DNA during coWGA. Consequently, the percentage of damaged cellular target DNA in single cells can be measured indirectly by determining the quantity of Control-DNA amplified during coWGA.

### Co-linearity of cell number and Control-DNA amplification

First, we tested the MDA concept using a competitive Control-DNA and a serial dilution of Jurkat cells. An amount of 0.6 pg of denatured Lambda DNA was spiked as a competitive Control-DNA during MDA. Although other amounts of Control-DNA may be useful too, we found a lower reproducibility by lowering the amount of Control-DNA. To avoid massive over-representation of Control-DNA after amplification, we did not spike higher amounts of Control-DNA. After MDA, real-time PCR was performed using coWGA amplified DNA and primers specific for Lambda Control-DNA. No-template-control coWGA reactions (NTC) that contain no cell but only Control-DNA resulted in Cq values of ~9.3 (+/−0.16). All other coWGA reactions containing at least a single cell generated Cq values significantly higher (at least 4 cycles higher) than the Cq value of the NTC reaction indicating that Control-DNA was outcompeted as template by cellular DNA during coWGA.

**Figure 2:**
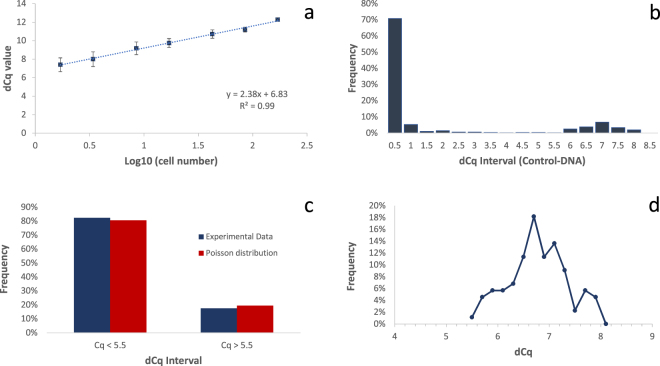
(**a**) Linear correlation of cell number and dCq value of Control DNA. After coWGA of various cell numbers, dCq is determined in real-time PCR. The dCq value of Control-DNA is plotted against the common logarithm (log_10_) of cell number. The standard deviation is given as error bars. The dCq value of co-amplified Control-DNA clearly correlates with the common logarithm of cell number used in WGA. (**b)** Frequency of WGA reactions characterized by dCq intervals of co-amplified Control-DNA: WGA was performed using Control-DNA and a cell dilution of 0.2 cells/WGA. As expected from Poisson distribution, most reactions did not contain any cell and resulted in a low dCq value. dCq values of ~7 represents WGA reaction containing at least a single cell. (**c)** Comparison of experimental data and Poisson distribution after WGA with 0.2 cells/WGA in average. WGA reaction with dCq value < 5.5 correlated with expected fraction of WGA reactions containing 0 cells. The second cluster of WGA reactions (dCq > 5.5) correlated with expected fraction of WGA reactions containing at least one cell. (**d)** Distribution of dCq value intervals of 0.2 cycles of WGA reactions containing cells.

In a next step, we calculated the delta-Cq (dCq) value by subtracting the Cq value of NTC WGA reactions from the Cq value of WGA reaction (containing a cell). Since we expected WGA reactions without cells, we only used reactions with cells characterized by Cq values significantly higher than determined from NTC reactions for the calculation. Figure 2a shows a high correlation (R^2^ = 0.99) between cell number and average dCq values of the competitive Control DNA after coWGA. Because the method measures the competitive amplification of cellular DNA and Control-DNA, the slope is positive and not negative as typically found for real-time PCR. In standard real-time PCR experiments determining a genomic marker with high efficiency, a 10-fold higher cell number results in a 3.3 lower Cq value, corresponding to the slope (m) of the line of best fit when cell numbers are given in common logarithm. Looking at the competitive amplification of Control-DNA in coWGA, we measured a Cq values that are 2.38 cycles higher when increasing the cell number 10-fold (slope in Fig. 2a). This is indicative of reduced amplification efficiency of Control-DNA during WGA in the presence of intact DNA from cells.

Because the genome of a single cell oscillates between non-replicated and replicated, the dCq value span can be calculated by y = m * log_10_(2) with the slope m of 2.38. Thus, a single cell can span 0.72 cycles from a nonreplicated to a replicated cell. According to Poisson distribution, the standard deviation is expected to increase with lower cell numbers, because relative cell number per well varies more widely at low cell numbers. As expected, we found a reciprocal relation of cell number and standard deviation (see error bars in Fig. 2a).

To calculate the apparent average cell number in the highest cell dilution, we determined the number of samples with a dCq value close to No-template-control coWGA reactions (NTC). For the highest cell dilution, we found Cq values close to the NTC reaction in about 19% of 48 coWGA reactions. For the second highest cell dilution, we determined that about 4% of the reactions resulted in Cq values close to the 9.3 cycles of the NTC samples. Using Poisson distribution calculation, the numbers of coWGA reactions without any cell indicated that the highest cell dilution contain an average cell number of ~1.7 cells per sample. In this case, 18.4% (highest cell dilution: 1.7 cells/sample) or 3.4% (second highest dilution: 3.4 cells/sample) WGA reactions would contain no cell, as calculated by Poisson distribution. To calculate the dCq value of a single cell, the dCq value obtained from the highest cell dilution (1.7 cells/sample) must be corrected. The highest dilution of cells resulted in a dCq value of 7.37 (see axial intercept; Fig. 2a). Using the formula above, we calculated a dCq value of a single cell by y = 7.37 − m * log_10_(1.7) using 1.7 as the corrected cell number. Thus, the corrected dCq value for one single cell would be 6.8 cycles. In summary, the competitive amplification of Control-DNA and cellular DNA during WGA showed an exponential dependency on cell number and can be used to simply discriminate between WGA reactions containing no target cell and WGA reactions with cells. A typical dCq value after WGA of competitive Control DNA and target DNA of one single cell under the conditions described is 6.8 cycles.

To experimentally determine the dCq value of a single cell, we started an WGA experiment with an average of 0.2 Jurkat cells per sample, generated by cell dilution and Control-DNA using 0.6 pg Lambda DNA/sample. 470 samples were used for WGA and subsequent real-time PCR. We calculated the dCq value of all samples by subtracting the Cq value of the NTC samples (here: 9.3 +/− 0.26) from the Cq value of the samples. In Fig. 2b, the dCq values are sorted according to their size intervals. Most of the WGA reactions resulted in dCq values below 1, followed by a very few reactions with dCq values between 1 and 5.5. A second group of WGA reactions clusters at dCq values greater than 5.5 (see Fig. 2b,c). The WGA reactions with a dCq value lower than 5.5 accounted for 82.4% of all reactions which is close to the expected value (81.9%) of the Poisson distribution for wells without cells if using a dilution of 0.2 cells per well. According to Poisson distribution, we expected ~16.4% of WGA reactions containing a single cell and 1.7% reactions containing at least two cells. Thus, the expected fraction of WGA reaction containing at least one cell is 18.1% which is close to the experimental value of 17.6%. As seen above, competitive amplification of Control-DNA and cellular DNA during WGA can be used to simply discriminate between WGA reactions containing no target cell and WGA reactions with cells. We analyzed the second cluster of WGA reactions characterized by dCq values higher than 5.5 in more detail. According to Poisson distribution, about 90% of cell-containing WGA reactions should comprise a single cell, if WGA was performed with an average of 0.2 cells/WGA, and only 10% of WGA reactions comprise two or more cells. We found that most WGA reactions peaked at dCq of ~6.7 (Fig. 2d). The mean dCq value of all WGA reactions within this cluster reaches also the value 6.7 cycles and is close to dCq value for one single cell determined above (dCq = 6.8). Therefore, we proposed that a dCq of ~6.7 corresponds to the average value of a single cell in this experimental setup.

### Identification of cells with damaged genomes by WGA with Control-DNA

Since a single cell spans a dCq interval of 0.72 cycles, the question remains how to explain WGA samples resulting in dCq values outside of the interval representing a single cell. It is obvious from Poisson distribution that higher dCq values represent WGA samples with higher cell number (see also Fig. 2a). According to Poisson distribution, about 10% of all samples with cells comprise more than a single cell if the average cell number is 0.2 cells/sample. If we define samples with a dCq value above 7.4 (= 6.7 + 0,72) as samples containing more than a single cell, we found that 87.5% of all positive samples comprise a single cell and 12.5% of all positive WGA samples contained more than a single cell. These values are in good correlation with expected values from Poisson distribution (90.3% and 9.7%, respectively). The slightly higher value for the 2-cell fraction is expected because a small fraction of Jurkat cells are polyploid^20^.

**Figure 3:**
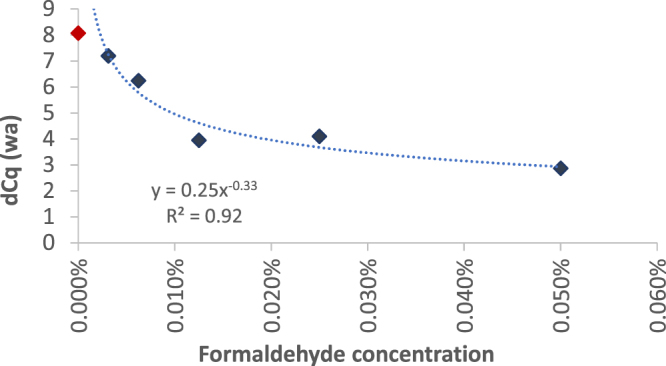
Correlation of dCq value (weighted average, wa) and formaldehyde concentration used for cell damaging. Various formaldehyde concentrations have been used to introduce different densities of blocking sites for DNA amplification. The weighted average was calculated from dCq value intervals of Control-DNA. The weighted average dCq value decreased exponentially by increasing the formaldehyde concentration indicating damaging of single cell DNA (see blue diamonds). The red diamond gives the weighted average dCq value of non-treated cells used as a control.

Although a clear majority of samples showing a significant difference to the NTC control resulted in a dCq value of ~6.7, which represents a single cell, a significant fraction of samples (here: ~35%) lead to lower dCq values in this experiment (see Fig. 2d; dCq < 6.7 cycles). We followed the hypothesis that these samples represent WGA reactions using a single cell with damaged genomes (e.g. apoptotic cells). In addition, we assumed that the dCq value correlates with the degree of damage. To test this hypothesis, we induced damage to HeLa cell DNA by formaldehyde treatment. HeLa cells were seeded and grown overnight. After cell harvesting and counting, cells were exposed to formaldehyde. Because cells are not part of a solid tissue sample, all cells are highly accessible for formaldehyde. Therefore, we chose low concentrations of formaldehyde up to 0.05% in PBS to introduce blocking sites in single cell DNA. After treatment, cells were counted and aliquoted with an average density of 1 cell/sample. After coWGA of Control-DNA and cellular DNA obtained from formaldehyde treated cells and real-time PCR, we determined dCq values between ~3 and 9.3. We calculated the weighted mean of dCq values for different formaldehyde concentrations. The weighted mean dCq decreased exponentially depending on the formaldehyde concentration. This is indicative for an increased degree of DNA damage in the single cell (Fig. 3). As a control, coWGA reactions were performed using one cell or 50 cells on average without formaldehyde treatment. For 50 cell samples, an average dCq value of 10.7 was determined. We calculated an expected dCq value of 10.75 by dCq = 6.7 + 2.38 * log_10_(50). In conclusion, the dCq value determined after coWGA of 50 cells is in good correlation with the expected value for this cell number.

**Figure 4:**
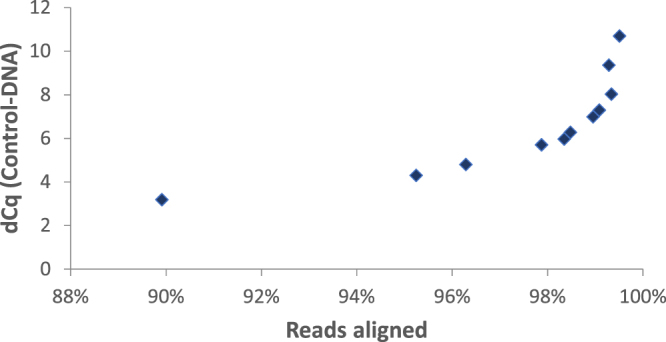
Fraction of aligned reads plotted over dCq value of Control-DNA after WGA. About 2.3 × 10^6^ reads are aligned per library to the human genome. The fraction of reads that are aligned to the human genome changes with the degree of damaging of cells. The dCq value of ControlDNA spiked during WGA of single human cells correlates exponentially with the fraction of the reads aligned to the human genome.

**Figure 5:**
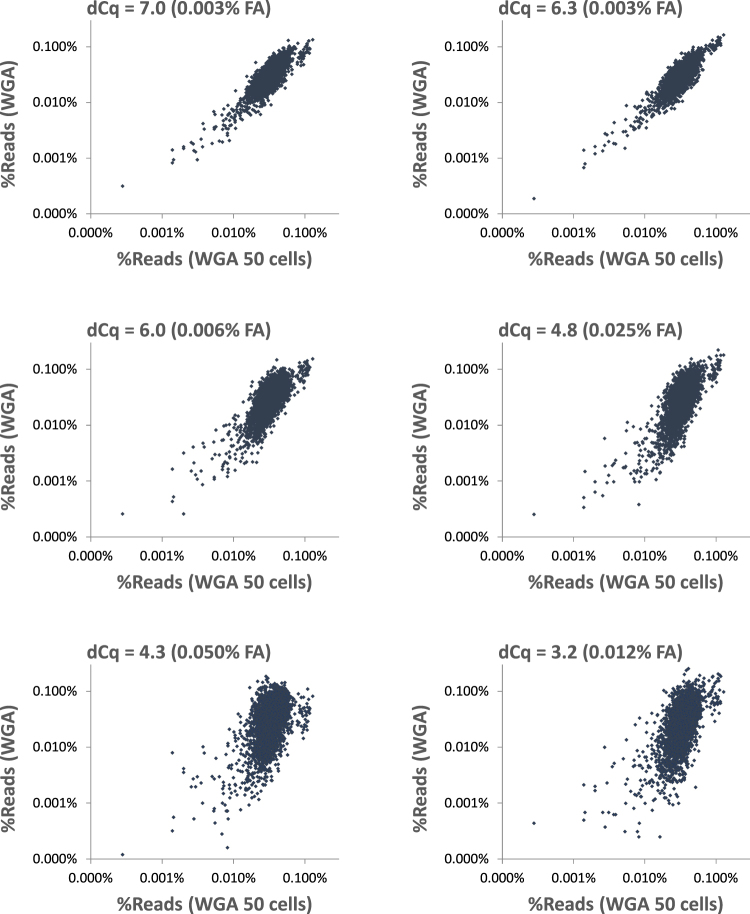
Single cell WGA vs WGA of 50 cells. The figures show scatter plots of relative read counts mapped to human genome 1 mbp bins after WGA sorted according to their dCq value of Control-DNA. On the X-axis relative read counts are represented of WGA reaction of 50 cell sample that were not treated by formaldehyde. On Y-axis, relative read counts are shown of samples with a single formaldehyde treated cell on average. The formaldehyde concentration (FA) is indicated. All WGA reactions were performed in the presence of competing Control-DNA. WGA reactions were selected according to their dCq value of Control-DNA for NGS (see headline of scatter plots). Deviation of read counts correlated with dCq value of Control-DNA after WGA and formaldehyde treatment. The higher the formaldehyde concentration is for DNA damaging within cells, the higher is the deviation of read counts and the lower is the dCq value. Exception is the WGA reaction characterized by a dCq value of 3.2 (last panel). Here, the formaldehyde concentration is intermediate but resulted in high deviations of read counts. It is most likely that this cell was already damaged prior to formaldehyde treatment. Therefore, an intermediate formaldehyde concentration is enough to damage the single cell DNA strongly. However, the dCq value is a good indicator for sequencing outcome also for this cell.

After real-time PCR analysis, we prepared genome libraries for NGS sequencing on a MiSeq instrument. On average 2.35 × 10^6^ reads were obtained from each library. The reads were mapped to the human genome and lambda phage genome (Control-DNA). The dCq value of Control-DNA correlated with the number of reads aligned to the human genome of the target cells. The fraction of reads aligned to the human genome increased exponentially with the increase of dCq value of control DNA (Fig. 4). Because dCq values lower 6.7 indicated damaged DNA within single cells (Fig. 3), we concluded the lower fraction of reads aligned to the human genome is a result of damaged DNA within single cells used for WGA. In contrast, we did not detect a significant change of the rate for errors, insertions, deletions or chimera formation among the samples with different dCq value (data not shown). Because blocking sites result in lower amplification of affected DNA regions, damaged cells result in less homogeneous genome representation after WGA. To visualize sequence representation after coWGA of formaldehyde damaged cells we plotted the relative read counts mapped to human genome bins against a coWGA amplified 50 cell sample. The plots were matched to the corresponding dCq values of Control-DNA. It is striking that a reduction of dCq of Control-DNA within single cell WGA DNA coincide with a higher deviation of the ideal diagonal in scatter plots (Fig. 5). Typically, the treatment of cells with higher formaldehyde concentration resulted in lower dCq values and higher deviation in read counts indicating more blocking sites in damaged DNA. Exceptions (see Fig. 5 second and last panel) are assumed to be a consequence of treating already damaged cells by formaldehyde (lower dCq value as expected by a dedicated formaldehyde concentration) or a higher number of damaged cells as the expected (higher dCq value as expected by a dedicated formaldehyde concentration, not shown). Single cell WGA samples with a dCq value > 6.3 resulted in read counts per bin that are very similar to 50 cell WGA reactions. A dCq value below 6.3 indicated higher variation in read counts compared to 50 cell WGA reactions. In conclusion, we found an increase in variation of genome marker representation with decreasing dCq value of Control-DNA. High variation of genome marker reads correlated with low DNA quality due to formaldehyde-induced lesions affecting WGA. Thus, the dCq value of Control-DNA is a good measure for DNA quality within single cells and for DNA quality of WGA amplified material.

**Figure 6:**
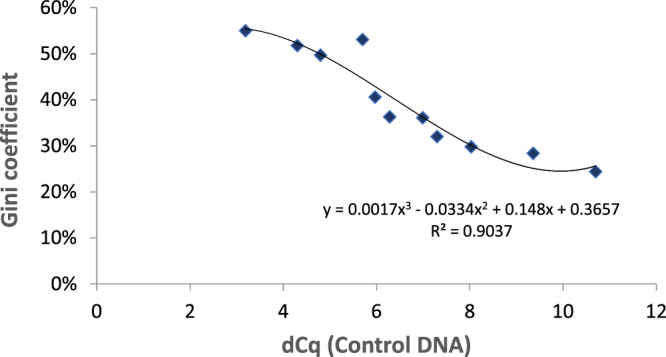
Correlation of dCq value of Control-DNA during coWGA and Gini coefficient. The Gini coefficient was determined from read counts mapped to 100 k bins of the human genome. While the dCq value is determined prior to NGS library formation and sequencing, the Gini coefficient is calculated after sequencing.

Our data indicated that damaged DNA resulted in unequal representation of genome sequences after WGA (Fig. 5). To further quantify the effects of damaged single cell DNA on WGA and sequencing, we calculated the Gini coefficient, a statistical measure for dispersion. The Gini coefficient is a value between 0 (maximal equality) and 1 (maximal inequality). Here, the Gini coefficient (Fig. 6) measured the extent of unequal sequence representation after WGA of single cells of different qualities. The read numbers per bin were used to calculate the Gini coefficient. We opposed Gini coefficient and dCq value of Control DNA in a scatter plot and found an inverse correlation (R^2^ = 0.89): Higher dCq value (increase in DNA quality of a single cell) correlated with lower Gini coefficient and vice versa. A low dCq value correlated with unequal sequence representation after coWGA reaction (high Gini coefficient), based on cells damaged by formaldehyde. Cells with a lower degree of damaging (high dCq values) resulted in a more homogeneous (unbiased) amplification during WGA (low Gini coefficient). In conclusion, the dCq value of the Control-DNA coamplified during single cell coWGA is a good proxy to predict unequal amplification induced by damaged DNA and can be determined directly after WGA and prior to expensive NGS library sequencing needed to calculate the Gini coefficient.

## Discussion

High DNA quality is important for the accuracy of genome analysis using e.g. microarrays or sequencing^13^. DNA alterations such as breaks, thymine dimers, or abasic sites stops DNA synthesis during polymerase dependent reactions. Therefore, DNA damage reduces robustness of results, increases standard deviation, and induces difficulties in interpreting data of genome analyses^21^. Also for whole genome amplification samples, the quality of genome data is highly dependent on the quality of primary target DNA^13,22^. The impact of DNA damage raised with decreasing DNA copy number making it a crucial factor in single cell analysis.

In case of single cells, DNA quality cannot be determined without consuming at least a fraction of the sample with current methods. Consequently, the quality of single cells cannot be tested and single cell genome analysis appears to be a blind flight up to the point when data are analyzed. Bioinformatic tools have been developed to uncover the quality of the data set after molecular analysis of the single cell genome, e.g. by the assessment of relative read counts per bin or by calculating the Gini coefficient after genome sequencing. In contrast to these bioinformatic tools, we introduce a new method that enables the qualification of single cells after whole genome amplification to enable high quality genome analysis. The new competitive whole genome amplification (coWGA) (Fig. 1) is based on the competitive amplification of spike-in Control-DNA and target single cell DNA. After coWGA, a simple real-time PCR of Control-DNA results in a dCq value which gives a good indication of the quality and quantity of single cell DNA and - therefore - of the resulting WGA DNA. Although the method is similar to competitive PCR^18,19^, coWGA cannot simply be transferred to PCR based WGA methods. The coWGA method described here is optimized to the needs of Phi29 polymerase, used for proofreading WGA methods (MDA).

We show that the dCq value of Control-DNA after coWGA can be used to select the right samples for single cell analysis such as cost-intensive and laborious sequencing methods. Thus, sequencing runs are avoided from unsuitable samples. Instead, those samples that are prequalified and which are used for sequencing will be of high quality. Therefore, genome sequencing generates higher value. The dCq value of Control-DNA can be taken to differentiate between WGA reactions containing cells and those without cells (Fig. 2b,c). Additionally, the dCq value correlated with the log_10_(cell number) and we could determine the dCq value for a single cell (Fig. 2d). Typically, we determined a dCq value of ~6.7 for a single human cell if using 0.6 pg of Control-DNA. We confirmed the data with other real-time cycler instruments from ThermoFisher (StepOne: ViiA7; 7500), Agilent (Mx3500P), or QIAGEN (Rotorgene) (data not shown). The coWGA method can easily be adapted to the needs and requirements of downstream applications. Using a single intact cell, the relative fraction of reads mapped to Control-DNA is about 1% of total reads. With damaged cells, the relative fraction Control-DNA reads increased with the damaging degree of cells (data not shown). If a reduced read number of Control-DNA is required (e.g. for cells with smaller genomes), the amount of Control-DNA during coWGA could be decreased carefully. In this case, the dCq values will change accordingly for a single cell and the dCq value must be redefined.

According to the concept, higher dCq values are associated with higher numbers of cells (Fig. 2a) or at least a higher amount of DNA. In contrast, lower dCq values correspond to less amplifiable target DNA (low quality DNA) if a single cell is used. DNA damages within cells are characterized by multiple polymerase stop sites such as double-strand breaks, thymine dimers, covalent crosslinking of DNA, or abasic sites. These polymerase stop-sites reduce the amplification rate of the target DNA, which in turn results in an over-amplification of the Control-DNA. We found that the dCq value correlated with the number of aligned reads (Fig. 4). Cells that are damaged by formaldehyde lead to dCq values significantly lower than ~6.7 cycles (dCq value of a single cell). This shows that the dCq is a good method to measure single cell genome damaging. In concert with lower dCq values, the deviation of read numbers mapped to genome bins and the Gini coefficient increases, indicating a WGA reaction biased by the damages within the single cell DNA (Figs 4 and 5). Interestingly, chimera formation is not increased by single cell DNA damaging. Lasken and Stockwell proposed a mechanism for chimera formation by which 3′-ends of displaced strands reanneal to single stranded DNA and are elongated by the polymerase^23^. Here, the increase of 3′-ends generated in damaged DNA due to polymerase stop sites on target DNA did not increase chimera formation. Beside the inconspicuousness in chimera formation, damaged DNA did not increase error rate or rate for insertion and deletions during coWGA. It is expected that base exchanges, deletions, or insertions did not happen as DNA damaging was performed by formaldehyde. Formaldehyde treatment results primarily in polymerase stop sites such as covalent crosslinks and abasic sites, but does not induce deletions, insertions, or base exchanges. Thus, our data indicate that an increase in DNA synthesis blocking sites did not affect proofreading activity of Phi29 polymerase.

As the typical dCq value of Control-DNA for an intact single cell is ~6.7, the dCq value of damaged cells is <6.7. In typical experiments, we selected WGA reactions with a dCq value > 6.5 for genome analysis by sequencing or real-time PCR. The number of coWGA reactions selected by dCq value varies. Among reactions with one single cell, we found typically 60–95% coWGA reactions with dCq values >6.5, depending on cell quality. Some uncertainty may be caused by measurement errors or by coWGA samples of two or more damaged cells that mimic the dCq value of a single non-damaged cell. We estimated the probability of coWGA of two or more damaged cells by the fraction of samples with dCq values < 6.5 and the expected fraction of samples with at least two cells.

For selected applications, the threshold can be adjusted to the needs of analysis. For some analyses, a less stringent threshold may be acceptable. An additional upper threshold may be required for other application e.g. to avoid multiple cells within a single WGA sample. Because amplification bias is a matter of DNA quality^13^, we assume that experiments describing high amplification bias during MDA is based on a lower DNA quality within single cells. For example, some single cell experiments may be performed with cells stored for a long time. Because storage of cells can damage genomic DNA to an extend that amplification bias may be introduced during WGA. In summary, the use of a competitive Control-DNA during single cell WGA results in a better standardization, is labor- and cost-saving and avoids the use of non-controlled samples for deep genome analysis.

## Conclusion

The quality of whole genome amplification depends on the quality of the starting material. We developed the competitive whole genome amplification (coWGA) for the amplification of single cell DNA with prospect to control DNA quality by a simple real-time PCR assay. The method allows the selection of suitable coWGA reactions for further analysis such as cost-intensive and laborious genome sequencing applications. In conclusion, the dCq value of the Control-DNA coamplified during single cell coWGA is in good agreement with bioinformatic analysis (e.g. the Gini coefficient) that can only be performed after sequencing. In contrast to bioinformatic tools, the dCq value can be determined directly after coWGA prior to expensive NGS library formation and sequencing.

## Material and Methods

### Cell culture

HeLa cells were grown in Gibco™ DMEM Medium (1x Dulbecco’s Modified Eagle Medium; ThermoFisher Scientific, Cat. No. 31885-023) including 10% Fetal Bovine Serum (Biochrom AG; Cat. No. S0415), 1% MEM NEAA (100x) (Minimum Essential Medium Non-Essential Amino Acid; ThermoFisher Scientific; Cat. No. 11140-035) and 1% Penicillin-Streptomycin (Sigma; Cat. No. P0781) overnight at 37 °C. Jurkat cells are grown in Gibco™ RPMI Medium 1640 (1x) (Thermo Fisher Scientific; Cat. No. 31870-025) including 10% Fetal Bovine Serum (Biochrom AG; Cat. No. S0415), 1% MEM NEAA (100x) (Minimum Essential Medium Non-Essential Amino Acid; ThermoFisher Scientific; Cat. No. 11140-035) and 1% Penicillin-Streptomycin (Sigma; Cat. No. P0781). After aspiration of medium, and PBS wash, HeLa cells are incubated in Trypsin/EDTA (Sigma; Cat. No. T3924) for 5–10 min at 37 °C followed by addition of medium to stop trypsination of cells. After centrifugation (Hettich Rotina 380 R; 5 min × 300 g) of HeLa or Jurkat cells and an additional washing in PBS, cells are counted in Counting Slides (BIO RAD; Cat. No. 145-0011) in TC20 Automated Cell Counter (BIO RAD) by staining with trypan-blue (BIO RAD; Cat. No. 145-0013). In general, a fraction of >80% of viable cells were determined. After counting, cells were diluted to the intended cell number per sample.

### Formaldehyde treatment of cells

We damaged HeLa cells by formaldehyde treatment. HeLa cells were seeded and grown overnight. After trypsin treatment, cells were harvested and counted. About 10^5^ cells were used for treatment with 0–0.05% formaldehyde in PBS for 5 min at room temperature. Treatment was stopped by adding medium, followed by centrifugation, resuspension in formaldehyde free PBS, and dilution to the intended cell number per sample.

### Whole genome amplification

For WGA, cells were diluted so that the intended cell number was suspended in a 4 μl volume. WGA was performed using the REPLI-g Single Cell Kit (QIAGEN; Cat. No. 150345) according to the protocol in the kit handbook. Briefly, 4 μl cell suspensions were incubated with 3 μl Buffer D2 for 10 min. After stopping the reaction by adding Stop Solution, a master mix comprising H20 sc, REPLI-g sc Reaction Buffer, and REPLI-g sc DNA Polymerase was added. The REPLI-g sc Reaction Buffer was spiked by 0.6 pg denatured Lambda DNA (Thermo Scientific; Cat. No. SD0011). Because Lambda DNA quality may have effects on the assay, it is advantageous to test different lots or samples of different vendors. The reaction was performed for 3 h at 30 °C. The concentration of the DNA after WGA was determined using the Quant-iT™ PicoGreen® dsDNA Reagent (Life Technologies, cat. no. P7581), according to the manufacturer’s instructions. The amount of Control-DNA was quantified by real-time PCR using Control-DNA specific primers.

### Real-time PCR

Real-time PCR amplification was performed by using 2 µl of 1/100 diluted WGA DNA, QuantiTect SYBR Green PCR kit (Cat. No. 204145) and the appropriate primers (final concentration: 0.5 µM each; lambda forward primer: gag acg ctg gag tac aaa cg; lambda reverse primer: cca gcg gat tat cgc cat act g). All primers were purchased from Integrated DNA Technologies (IDT). Typically, thermocycling was performed on an ABI ViiA7 real-time cycler instrument for 15 minutes 95 °C (initial denaturation) and 40 cycles for 15 seconds at 95 °C, 30 seconds at 56 °C by 30 seconds 72 °C, followed by a melting curve analysis. The Cq value was determined for each sample. We tested other real-time PCR thermocycler instruments resulting in similar values which do not change the result significantly.

### Sequencing

For DNA fragmentation, we used 2 μg genomic DNA or WGA DNA (without subsequent purification of WGA DNA) and the Covaris® S220 instrument. After fragmentation, DNA was purified using the MinElute® PCR Purification Kit (QIAGEN) according to the protocol in the kit handbook. Library preparation was performed using either the GeneRead™ Library Prep Kit (I) (QIAGEN) according to the manufacturer’s instructions. The NGS library was purified using the GeneRead Size Selection Kit (QIAGEN) as per instructions in the kit handbook. We performed 150 bp paired-end sequencing on an Illumina® MiSeq® instrument.

### Data Analysis

For reference mapping, we used the complete human genome data of Homo sapiens (assembly GRCh38). Read alignment was performed using BWA MEM^24^. The coverage in bins of 1 mbp size was determined using BEDTools^25^ (see also Fig. 5). To assess the equality of the read-distribution we calculated the Gini coefficient across 100 kb genomic bins using the R-package ineq^26^ (see Fig. 6). The Gini coefficient measures the inequality among values of a frequency distribution (depth of coverage in this case). A Gini coefficient of zero expresses perfect equality, where all values are the same. A Gini coefficient of one (or 100%) expresses maximal inequality among values.

### Data availability statement

With reference to this publication data are available via email: Christian.Korfhage@qiagen.com.
